# Exercise preconditioning and resveratrol reduce the susceptibility of rats with obstructive jaundice to endotoxin and alleviate lung injury

**DOI:** 10.3389/fimmu.2024.1466615

**Published:** 2024-12-18

**Authors:** Xujiong Li, Wei Li, Tao Wang, Yan Li, Li Zhao

**Affiliations:** ^1^ Department of Exercise Physiology, Beijing Sport University, Beijing, China; ^2^ Department of Physiology, Changzhi Medical College, Changzhi, China; ^3^ School of Pharmacy, Changzhi Medical College, Changzhi, China; ^4^ Key Laboratory of Physical Fitness and Exercise, Ministry of Education, Beijing Sport University, Beijing, China

**Keywords:** obstructive jaundice, endotoxemia, inflammation, lung injury, exercise, resveratrol

## Abstract

**Introduction:**

Endotoxemia is a common issue for patients with biliary obstruction. The lung is the most affected organ by endotoxins. Exercise training can alleviate lipopolysaccharide (LPS)-induced lung inflammation and resveratrol has biological effects similar to exercise. In this study, we evaluated the protective effects of exercise preconditioning, resveratrol, and their combination on LPS-induced lung injury and mortality in rats with obstructive jaundice.

**Methods:**

Endotoxemia was simulated in rats by common bile duct ligation (CBDL) and intraperitoneal injection of low-dose LPS. The treatment groups were pretreated with exercise and/or resveratrol to assess their effects on lung injury and mortality. Immunohistochemistry, immunofluorescence, and ELISA were subsequently used to evaluate the impact of exercise and/or resveratrol on inflammation in lung tissue and bronchoalveolar lavage fluid.

**Results:**

We found that even in the early stages, compared to sham-LPS rats, low-dose LPS induced excessive systemic inflammatory responses in CBDL rats, as evidenced by a significant increase in TNF-α and IL-6, severe lung inflammation, lung injury, and higher mortality rates, indicating that cholestasis increased rats’ susceptibility to endotoxins. Exercise training reduced neutrophil infiltration in the lungs of model rats and IL-6 levels in bronchoalveolar lavage fluid. Both exercise training and resveratrol exhibited synergistic effects in reducing macrophage accumulation in lung tissues, lowering TNF-α and IL-6 levels in the lungs, and decreasing TNF-α concentration in bronchoalveolar lavage fluid. Additionally, exercise and combined interventions both significantly increased the expression of IL-10. The interventions induced a marked improvement in lung tissue pathological damage and lung edema in model rats and prolonged the survival time of rats with obstructive jaundice.

**Discussion:**

This study demonstrates that exercise preconditioning and/or resveratrol can significantly reduce rats’ susceptibility to endotoxins after CBDL and alleviate lung injuries through their anti-inflammatory effects, thereby decreasing the mortality risk.

## 1 Introduction

In clinical practice, endotoxemia is a common issue in patients with obstructive jaundice (1). It has been reported that during cholestasis, 50%-70% of patients can develop endotoxemia even in the absence of a clear source of infection (2). In such cases, the combination of various potentially cytotoxic bile components and the enhanced susceptibility of obstructive jaundice patients to endotoxins often lead to severe tissue and organ damage (2–4). The lungs are the most affected organs by endotoxins. Studies have shown that even low doses of lipopolysaccharide (LPS) can induce severe inflammatory reactions and lung damage in animals with obstructive jaundice (5, 6). This not only complicates the management of the underlying condition but also significantly reduces the survival rate of patients. Thus, investigating the molecular mechanisms underlying lung injury in patients with obstructive jaundice and endotoxemia, as well as exploring relevant preventive and therapeutic strategies, holds considerable clinical importance.

The combination of common bile duct ligation (CBDL) with low-dose LPS intraperitoneal injection simulates the clinical features of patients with biliary obstruction developing intestinal endotoxemia or other perioperative infections, making it a common method to induce the combined model of obstructive jaundice and endotoxemia in animals (7–10). In such models, cholestasis enhances the susceptibility to endotoxins, leading to excessive inflammatory reactions in animals (2, 11, 12). Under this condition, with the exacerbation of systemic inflammatory responses, the levels of circulating TNF-α and IL-6 increase, leading to the massive activation and accumulation of monocytes/macrophages and neutrophils in lung tissues, promoting the production of large amounts of reactive oxygen species, reactive nitrogen species, proinflammatory cytokines, proteases, etc., and, causing severe damage to lung tissues (5, 13, 14). Therefore, the use of anti-inflammatory therapeutic drugs, or the development of strategies to enhance the body’s resistance to endotoxins and inflammation before the disease triggers the aforementioned changes, are important intervention measures to alleviate lung damage.

Studies have demonstrated that long-term moderate and regular exercise training can significantly enhance the body’s anti-inflammatory capacity by regulating the immune system, and has demonstrated positive effects in the prevention and treatment of various lung diseases (15–17). In animals with non-obstructive jaundice, exercise training can alleviate chronic obstructive pulmonary disease, asthma, lung ischemia-reperfusion, and lung inflammation and damage induced by endotoxin and bacteria by altering the numbers of various immune cells (18–22). Additionally, exercise training can also exert a protective effect on lung injury by increasing anti-inflammatory cytokines (such as IL-10) and reducing the expression of pro-inflammatory factors (23, 24). A common feature of these disease models is that LPS is a significant cause of lung inflammation and damage. Considering that exercise training can alleviate LPS-induced lung inflammation, it can be reasonably speculated that exercise may also have a protective effect on endotoxemia in CBDL rats. Furthermore, the plant-derived polyphenolic compound resveratrol (RSV) has similar biological effects to exercise training and is often used as an exercise mimic (25, 26). Studies have shown that resveratrol and exercise training can activate the same signaling pathways, enhancing cellular energy metabolism and boosting immune function, thereby improving overall health (25–27). From an inflammatory perspective, RSV also exhibits significant anti-inflammatory properties. It can alleviate inflammatory responses by inhibiting the accumulation of inflammatory cells and suppressing the expression and release of pro-inflammatory cytokines, such as TNF-α and IL-6. Furthermore, studies on certain respiratory diseases have confirmed that RSV, similar to exercise training, can mitigate inflammatory damage to lung tissue through its anti-inflammatory effects. These findings suggest that their combined use may potentially enhance this anti-inflammatory effect and further reduce lung tissue damage.

Therefore, in this study, we established an animal model by administering LPS to CBDL rats and evaluated the protective effects of exercise preconditioning and/or RSV in this model, hoping to provide a theoretical basis for further clinical applications.

## 2 Materials and methods

### 2.1 Study animals

Healthy male SD rats (3 weeks old) were purchased from SPF (Beijing) Biotechnology Co., Ltd. (China), with License Number SCXK (Jing)2019-0010 and Experimental Use License Number SYXK (Jin)2022-0001. All animals were kept under a constant temperature (22-24°C) and maintained on a regular light-dark cycle. They were allowed ad libitum access to standard feed and tap water. All procedures in this study were conducted following the Laboratory Animal Care and Use Guidelines of Beijing Sport University, and were approved by the Ethics Committee of Beijing Sport University. Every effort was made to minimize the suffering of animals and the number of animals used.

### 2.2 Reagents

LPS (E. Coli 055:B5) and RSV were purchased from Shanghai Yuanye Bio-Technology Co., Ltd (Shanghai, China). The detection kits of total bilirubin (TBIL), alanine aminotransferase (ALT), and aspartate aminotransferase (AST) were obtained from Nanjing JianCheng Bioengineering Institute (Nanjing, China). The TNF-α and IL-6 ELISA kits were provided by AB Clonal Technology Co., Ltd (Wuhan, China). Recombinant Rat IL-6 (rrIL-6), BCA protein concentration assay kit (enhanced), and chemiluminescence reagents were from Beyotime Biotechnology Co., Ltd (Shanghai, China). Rabbit polyclonal antibodies against TNF-α and IL-6 were obtained from Proteintech Group, Inc (Wuhan, China), while the anti-IL-10 rabbit polyclonal antibody was from BEIJING BIOSYNTHESIS BIOTECHNOLOGY CO., LTD. (Beijing, China). CD68 mouse monoclonal antibody, Myeloperoxidase (MPO) rabbit monoclonal antibody, goat anti-mouse IgG H&L (Alexa Fluor 647), and goat anti-rabbit IgG H&L (FITC) were provided by Abcam. The goat anti-rabbit IgG (HRP) and immunohistochemistry kits were sourced from Zhongshan Golden-bridge (Beijing, China).

### 2.3 Experimental protocol

The animal model was established by performing CBDL and administration of LPS. To determine the optimal dosage of LPS and the experimental observation period, a sham group and a CBDL group were set up in the preliminary experiments according to the previous description (2, 28). At 80 h after surgery, based on the clinical manifestations and biochemical indicators of liver function, the sham group was administered with LPS at a dosage of 7.0 mg/kg through intraperitoneal injection, while LPS at dosages of 7.0 mg/kg, 5.0 mg/kg, and 3.0 mg/kg were respectively administered to CBDL rats. The survival status of each group was observed. The optimal dosage of LPS and the experimental observation period after LPS administration were determined as 3.0 mg/kg and 4 h, respectively.

**Figure 1:**
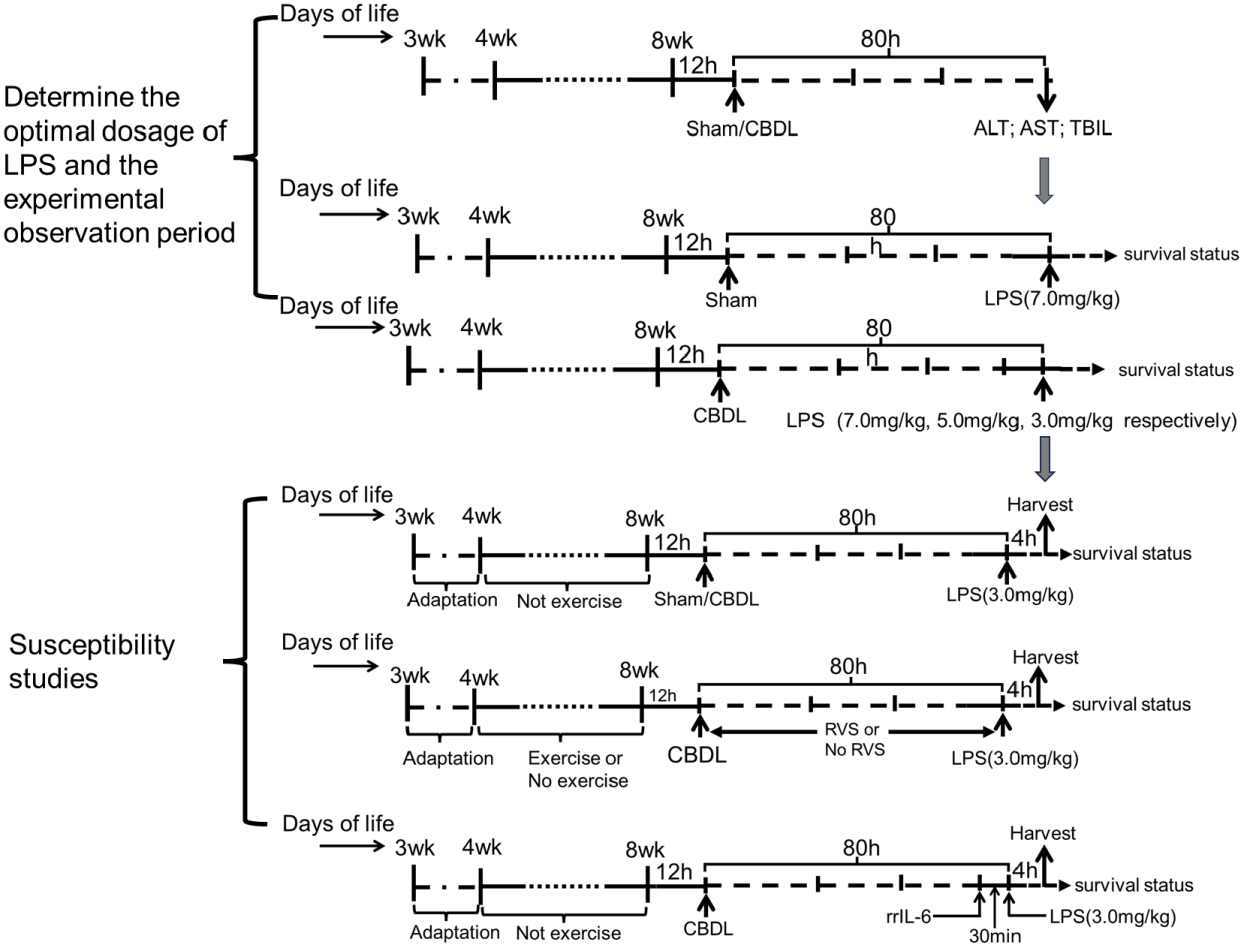
Schematic flow chart and timeline of the study design.

Based on preliminary experiments, in the second part of the study, CBDL rats were given 3.0 mg/kg LPS, and the mortality after corresponding interventions was observed. Specifically, the sham+LPS group, CBDL+LPS group, and CBDL+LPS intervention group were set up. The interventions in each group were exercise training, RSV, or their combination. Briefly, all rats underwent 1 week of exercise acclimation. Subsequently, the rats underwent 4 weeks of moderate-intensity exercise training. At 12 h after the training intervention, CBDL was performed. An intraperitoneal injection of LPS was conducted at 80 h after CBDL. RVS was prepared as a suspension in 0.5% carboxymethyl cellulose sodium and administered by gavage after CBDL (50 mg/kg, once daily), with this dosage determined based on previous studies (29, 30). The remaining groups were given an equal volume of 0.5% carboxymethyl cellulose sodium by gavage (**Figure 1**).

In the third part of the study, rats were randomly divided into the sham+LPS, CBDL+LPS, and CBDL+LPS intervention groups. The intraperitoneal injection of LPS (3.0 mg/kg) was performed at 80 h after surgery. After 4 h, specimens were collected to assess lung injury. The interventions, as mentioned earlier, included exercise, RSV, or their combination.

Additionally, to clarify the specific role of IL-6 in lung injury, we set up the CBDL-LPS group and the IL-6 intervention group in CBDL-LPS rats in the fourth part of the study. The rrIL-6 (2 μg/kg) was administered via tail vein injection 30 minutes before the LPS injection. The dose and administration time of rrIL-6 were determined previous description (31). The control animals were given an equivalent volume of normal saline.

### 2.4 Surgery procedures for CBDL

Twelve hours after the final exercise preconditioning, rats (330 g - 370 g) were anesthetized with isoflurane inhalation. Under sterile conditions, a midline incision of about 1.5cm was made below the xiphoid process of the abdomen, exposing and freeing the common bile duct. The common bile duct was doubly ligated using a 4/0 silk suture, and after cutting the common bile duct between the two ligatures, the abdomen was closed layer by layer. Sham-operated rats had their common bile duct exposed but not ligated, while the rest of the procedures were the same as those for the animals receiving CBDL. After surgery, the rats were closely monitored for breathing, heart rate, and body temperature. Once fully awake, they were housed in separate cages. Throughout the wound recovery process, regular observations and disinfection were conducted to ensure there are no infections.

### 2.5 Exercise training program

The exercise training was conducted according to the previous description, with minor modifications (21, 32). Briefly, the treadmill was set at zero incline for all stages of training, and all rats underwent adaptive training for 1 week. After this period, only those rats that actively participated in the exercise, performed well during the sessions, and demonstrated good recovery post-exercise, thus showing good adaptability, were selected for the subsequent 4 weeks of moderate-intensity exercise training. The training program for the rats started at 9 PM each evening, as detailed in **Table 1**.

**Table 1:** The continuous exercise training programs at different weeks.

Weeks	Duration/day(min)						Speed (m/min)
	D1	D2	D3	D4	D5	D6	
**First week (50% *v*_max_)**	20	22	24	26	28	30	12
**Second week (60% *v*_max_)**	32	34	36	38	40	42	15
**Third week (70% *v*_max_)**	44	46	48	50	52	54	18
**Fourth week (75% *v*_max_)**	56	58	60	60	60	60	21

### 2.6 Blood and bronchoalveolar lavage fluid (BALF) collection

The rats were anesthetized and fixed on the experimental table. First, the blood was collected from the abdominal aorta through an incision in the abdomen. Then, the chest was opened at the midline of the sternum. After exposing the trachea, the right main bronchus was clamped. A catheter was inserted into the left main bronchus, and 2.5 mL of phosphate-buffered saline (PBS) was instilled into the bronchoalveolar cavity of the left lung, followed by the collection of BALF. Each animal’s left lung underwent lavage three times.

### 2.7 Lung wet/dry ratios

The lower lobe of the right lung was collected, and its wet weight was measured after absorbing the surface moisture with absorbent paper. Then, the lower lobe of the right lung was placed in a drying oven at 80°C for 24 h. After that, the dry weight was determined. Finally, the wet/dry weight ratio of lung tissue was calculated.

### 2.8 Biochemical analysis

Blood samples were centrifuged at 4°C and 3000 r/min for 15 min to separate the plasma. The levels of AST and ALT were detected using a fully automatic biochemical analyzer. The total bilirubin (T-BIL) levels were measured according to the kit instructions on a microplate reader.

### 2.9 Analysis of cells in BALF

The BALF samples were centrifuged at 4°C at 3000 r/min for 10 minutes. The supernatant was collected, and the precipitate was resuspended in PBS (1 mL). A portion of the cell suspension was instilled on a slide for the Wright-Giemsa staining. The samples were subsequently then observed under a high-power microscope at 400x magnification. Five independent and non-overlapping fields of view were selected to count neutrophils and macrophages.

### 2.10 Quantitative detection of pro-inflammatory cytokines

The levels of pro-inflammatory cytokines TNF-α and IL-6 in plasma and BALF were quantitatively detected using the corresponding ELISA kits following the manufacturer’s instructions. IL-10 was also detected in BALF using the same method. Additionally, lung tissue homogenate at a concentration of 10% was prepared. and centrifuged at 4°C, 3000 rpm for 10 min. The supernatant was used for the ELISA analysis of TNF-α, IL-6, and IL-10 levels. Protein quantification was performed utilizing the BCA (Bicinchoninic Acid) method.

### 2.11 Histopathological evaluation

The left lungs were collected from another six rats of each group, fixed with 4% paraformaldehyde, dehydrated routinely, embedded in paraffin, and sliced to a thickness of 5 μm. Hematoxylin and eosin (HE) staining was performed, and pathological changes were observed under a light microscope. The lung injury scores were assessed based on the previous description, with appropriate modifications. Briefly, several parameters were evaluated in the histological sections from five random fields: A) inflammatory cell infiltration; B) tissue edema; C) thickening of alveolar septa; D) destruction of alveolar structures; and E) erythrocyte extravasation. Each parameter was scored from 0 to 4, where 0 indicates no alterations and 4 indicates most severe alterations. The total histology score was calculated as the sum of the scores for all parameters.

### 2.12 Immunohistochemistry

The 4 μm lung tissue sections were subjected to routine deparaffinization, rehydration, and inactivation of endogenous peroxidase with 3% H2O2 at room temperature for 15 min, followed by antigen retrieval. After washing with PBS, the sections were blocked with goat serum for 30 min. Subsequently, primary antibodies including rabbit anti-IL-10, rabbit anti-TNF-α, and rabbit anti-IL-6 were added, and the sections were incubated overnight at 4°C. This was followed by the addition of secondary antibodies and a 1 h incubation at room temperature. Following color development using DAB reagent and counterstaining with hematoxylin, the sections were dehydrated, cleared, and examined under the microscope after cover slipping. The brown-yellow particles represented positive staining. The semi-quantitative analysis was conducted using ImageJ software.

### 2.13 Immunofluorescence

Lung tissue sections (4 μm) were dewaxed and rehydrated routinely, followed by antigen retrieval and PBS washing. The sections were then blocked with 5% bovine serum albumin for 20 min. Primary antibodies against CD68 (1:400) and MPO (1:200) were added separately and incubated overnight at 4°C. The next day, after a 60-min rewarming period, species-appropriate fluorescent secondary antibodies were added, and the sections were incubated at room temperature for 1 h. After incubating with the DAPI mounting medium, the sections were examined under a microscope and analyzed using ImageJ software. The results were expressed as fold changes compared to the Sham-LPS group.

### 2.14 Statistical analysis

Statistical analysis was performed using SPSS 23.0 software. Normally distributed data are presented as means ± SD. The t-test was used to compare two groups of normally distributed data. Differences among multiple groups were analyzed using one-way analysis of variance (ANOVA), and after confirming the homogeneity of variances, the least significant difference method (LSD) was used for pairwise comparisons. Kaplan-Meier method was utilized for analyzing survival time and survival rate, and statistical significance was assessed by the Log-Rank test. A P value less than 0.05 was considered statistically significant.

## 3 Results

### 3.1 Model establishment

**Figure 2:**
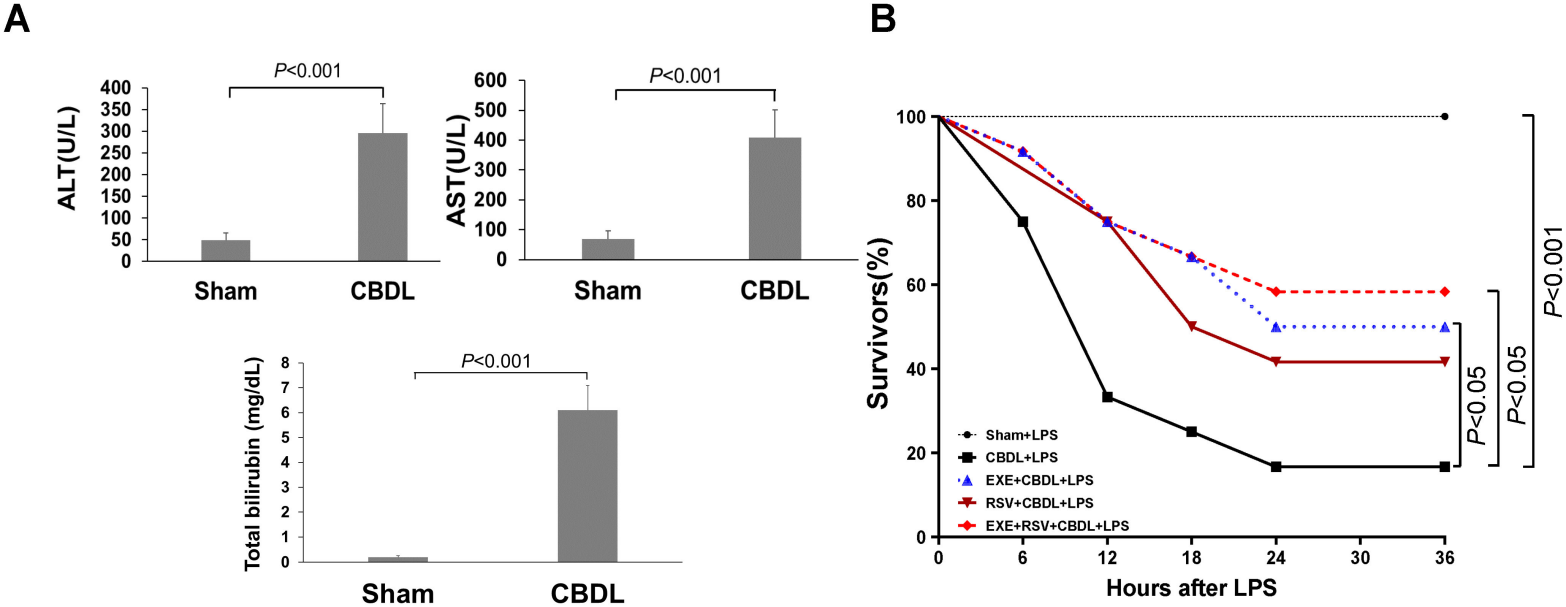
Model establishment and survival rate. **(A)** Changes in liver function markers in rats after CBDL (n=6). Data were analyzed by t-test. **(B)** Effects of exercise and/or RSV on the mortality rates of each group of rats (n=12). Survival Analysis, Kaplan-Meier and Log-Rank Test. LPS, Lipopolysaccharide; CBDL, common bile duct ligation; Sham, Sham operation; RVS, resveratrol; EXE, exercise.

On the first day after CBDL, the color of urine started to turn yellow. By day 3-4, jaundice was observed in the cornea, ear skin, and tail of the rats. The color of feces became lighter and clay-colored. These observations suggest the presence of cholestasis in rats after CBDL. Further analysis of blood biochemical indicators revealed a significant increase in ALT activity, AST activity, and TBIL content in the plasma of rats after CBDL (**Figure 2A**), indicating the successful replication of biliary obstruction.

To simulate the clinical conditions commonly associated with endotoxemia and potential sepsis during disease progression, we administered intraperitoneal LPS injections to different groups of rats. Preliminary experimental results showed that within 6 h after receiving 7 mg/kg of LPS, all sham-operated rats survived, while CBDL rats had a mortality rate of 83.33% (n=6). The mortality rate of CBDL rats receiving 5 mg/kg LPS was 66.67% within 6 h (n=6). CBDL rats receiving 3 mg/kg of LPS had a mortality rate of 25% within 6 h (n=12). The death of rats occurred between 4 to 6 h after LPS administration.

Given the high mortality rate of CBDL rats after LPS administration, we administered a single dose of 3 mg/kg LPS intraperitoneally for 4 h in all subsequent experiments.

### 3.2 General condition and survival rate

CBDL animals have high sensitivity to low-dose endotoxin. Before the administration of LPS, CBDL rats showed good survival status (100% survival), with normal activity and stable respiration. After intraperitoneal injection of 3 mg/kg LPS, sham-operated rats survived well with no obvious signs of respiratory distress. In contrast, CBDL rats exhibited slow movement, shortness of breath, and lethargy after approximately 1.5 h. Following the interventions of exercise and/or RSV, the general condition of CBDL rats was improved.

Survival analysis (n=12) revealed that the mortality rates of model rats were 25% at 6 h post-LPS administration, 66.67% at 12 h, and as high as 75% at 18 h. However, after the corresponding interventions, the mortality rates of the group receiving exercise training alone were 8.33% at 6 h, 25% at 12 h, and 33.33% at 18 h, as shown in **Figure 2B**. This indicates that the survival rate of rats after exercise training was superior to that of model rats, and simple exercise training showed a good intervention effect comparable to the combination of RSV and exercise training.

### 3.3 Pathological changes in lung tissue and lung wet/dry weight ratio

**Figure 3:**
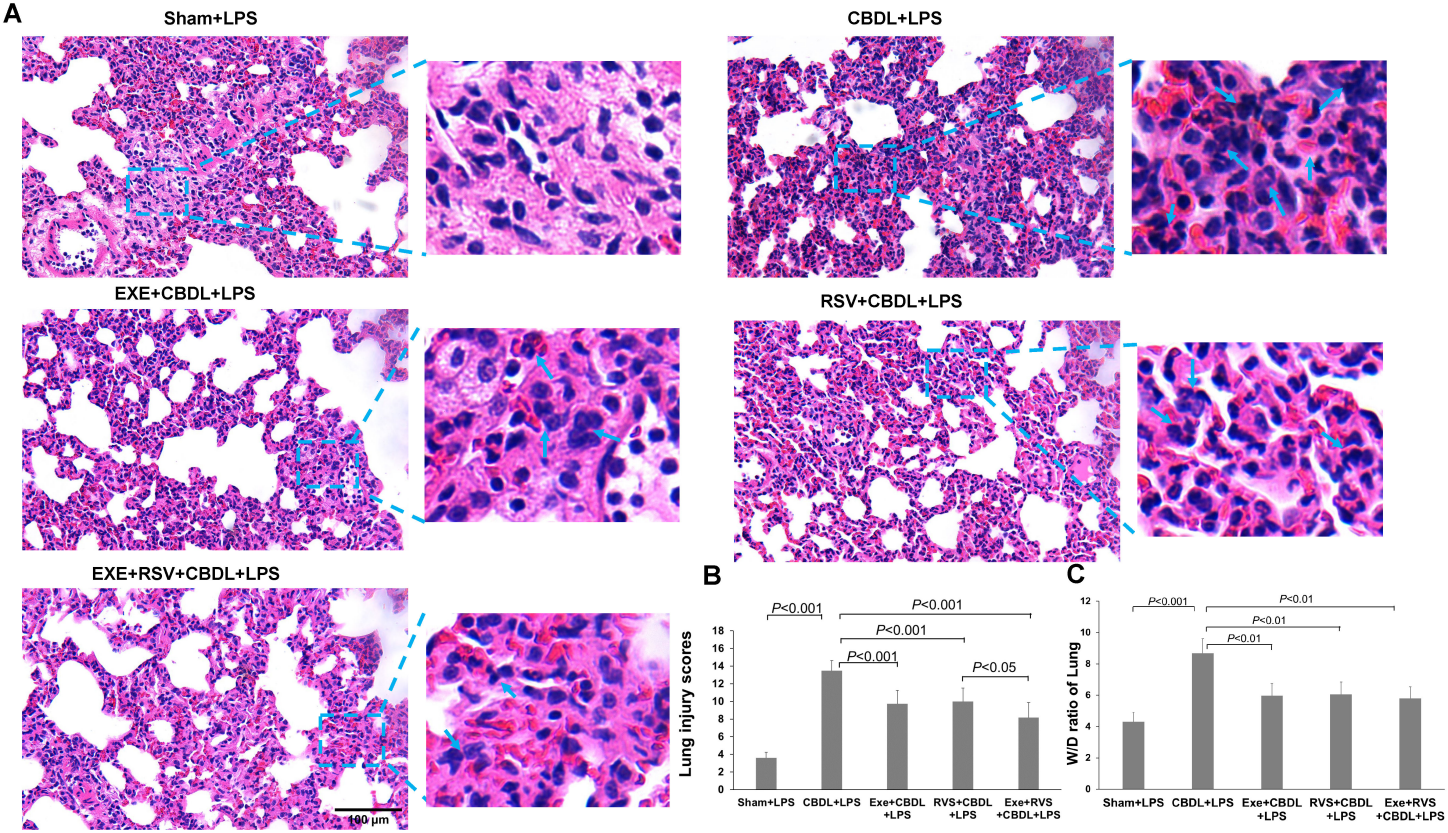
Pathological changes in lung tissue and lung wet/dry weight ratio (n=6). Arrows indicate significant accumulation of inflammatory cells and pronounced inflammatory response. **(A)** Representative HE staining images showing the pathological changes in lung tissue of each group. **(B)** Lung injury scores in each group. **(C)** Wet/dry weight ratio of lung tissue in each group. Data analyzed by one-way ANOVA, LSD test for group. LPS, Lipopolysaccharide; CBDL, common bile duct ligation; Sham, Sham operation; RVS, resveratrol; EXE, exercise; W/D, wet/dry.

Lung tissue is highly sensitive to LPS. To further confirm the increased susceptibility to LPS and the corresponding intervention effects after CBDL, we conducted a histopathological examination of lung tissue. HE staining analysis showed that low-dose LPS exposure could cause severe lung tissue damage in CBDL rats (**Figure 3A**). The main manifestations were narrowed alveolar cavities, evidently increased thickness of alveolar septa, and obvious infiltration of inflammatory cells in the lung interstitium. In comparison, the pathological changes in the intervention and sham groups were relatively mild. The alveolar structure of the sham group was intact, with clear alveolar activity and no obvious thickening of alveolar septa, but there was still evidence of inflammatory cell infiltration. In the intervention groups, the thickening of alveoli and damage to alveolar walls were reduced, inflammatory cell infiltration decreased, and red blood cell extravasation was improved. The improvement in lung injury scores is depicted in **Figure 3B**. Furthermore, the degree of chromatin margination in all intervention groups was reduced, compared to the CBDL-LPS group. In addition, the lung wet/dry weight ratio in the CBDL-LPS group significantly increased, indicating the occurrence of lung edema. Compared to the CBDL-LPS group, this ratio was significantly reduced in the intervention groups (**Figure 3C**), indicating an improvement in the degree of lung edema and damage.

### 3.4 Effect of exercise and/or RSV on inflammatory cell infiltration in lung tissues

**Figure 4:**
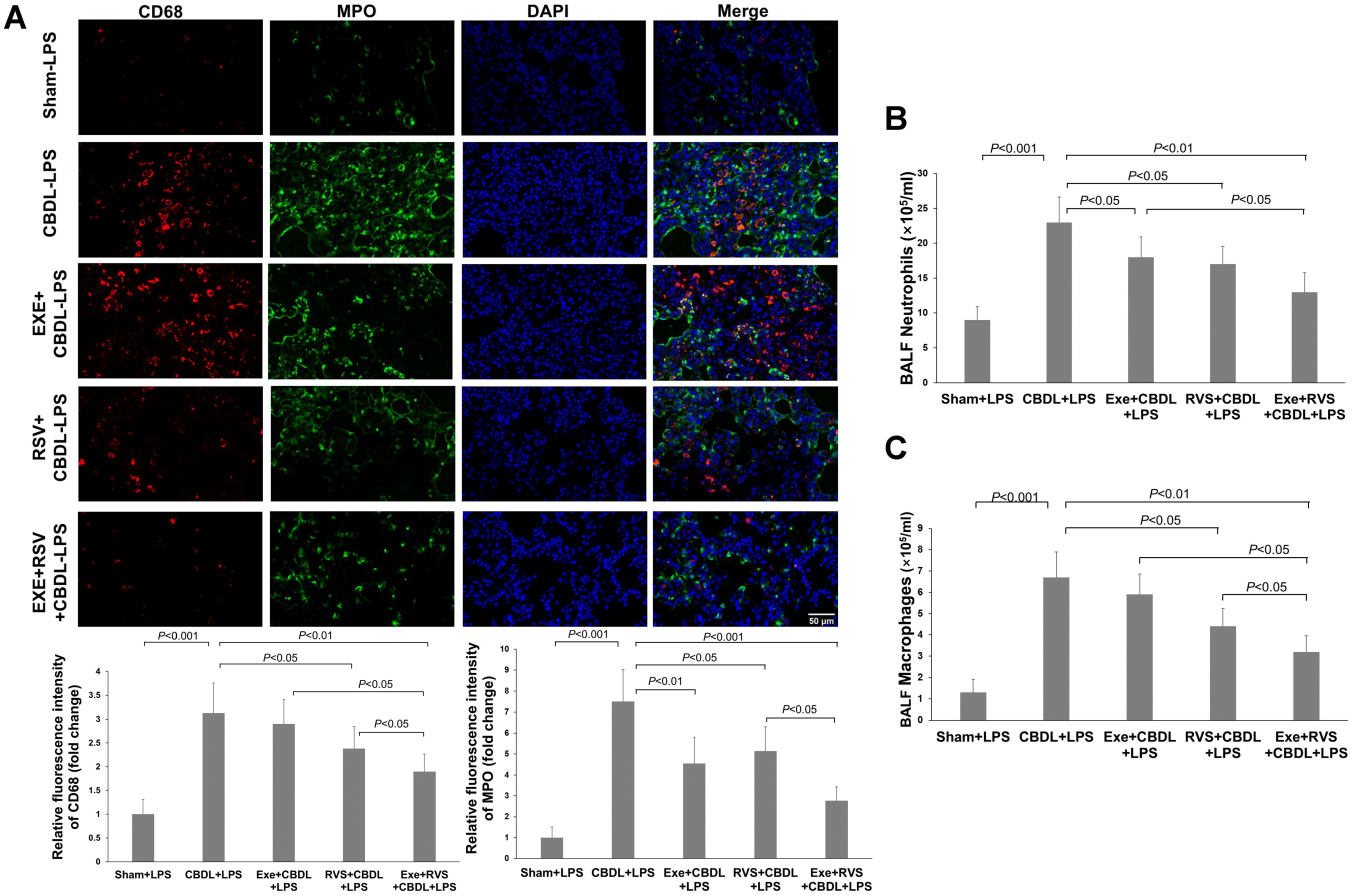
Effects of exercise and/or RSV on neutrophils and macrophages (n=6). **(A)** Representative immunofluorescence images of MPO and CD68. **(B)** Neutrophil counts in BALF. **(C)** Macrophage counts in BALF. Inflammatory cells in BAL fluid were counted under a 400x high-power microscope after Wright-Giemsa staining. Data analyzed by one-way ANOVA, LSD test for group comparisons. MPO, Myeloperoxidase; LPS, Lipopolysaccharide; CBDL, common bile duct ligation; Sham, Sham operation; RVS, resveratrol; EXE, exercise.

Neutrophils and macrophages are important inflammatory cells in the lung tissue of rats after CBDL. MPO can serve as an indicator enzyme for neutrophils, but it is also present in mononuclear/macrophages. Our immunofluorescence results revealed that after LPS administration, there was a significant increase in MPO and CD68 expressions in the lung tissue of the model rats, indicating a significant infiltration of neutrophils and macrophages. Exercise preconditioning alone, although not significantly affecting CD68-positive cells, significantly reduced the increase in MPO single-positive cells. Moreover, exercise training also enhanced the attenuation effect of RSV on CD68-positive cells (**Figure 4A**).

To further validate the changes in inflammatory cell infiltration, we evaluated the number of neutrophils and macrophages in BALF. The results showed that compared to the sham-LPS group, the levels of neutrophils (**Figure 4B**) and macrophages (**Figure 4C**) in the BALF of CBDL-LPS rats were significantly increased (P<0.001). Compared to the CBDL-LPS group, the respective interventions all reduced the number of neutrophils in the BALF of CBDL rats, and RSV and exercise combined with RVS also decreased the number of macrophages.

### 3.5 Effects of exercise and/or RSV on inflammatory and anti-inflammatory cytokines

**Figure 5:**
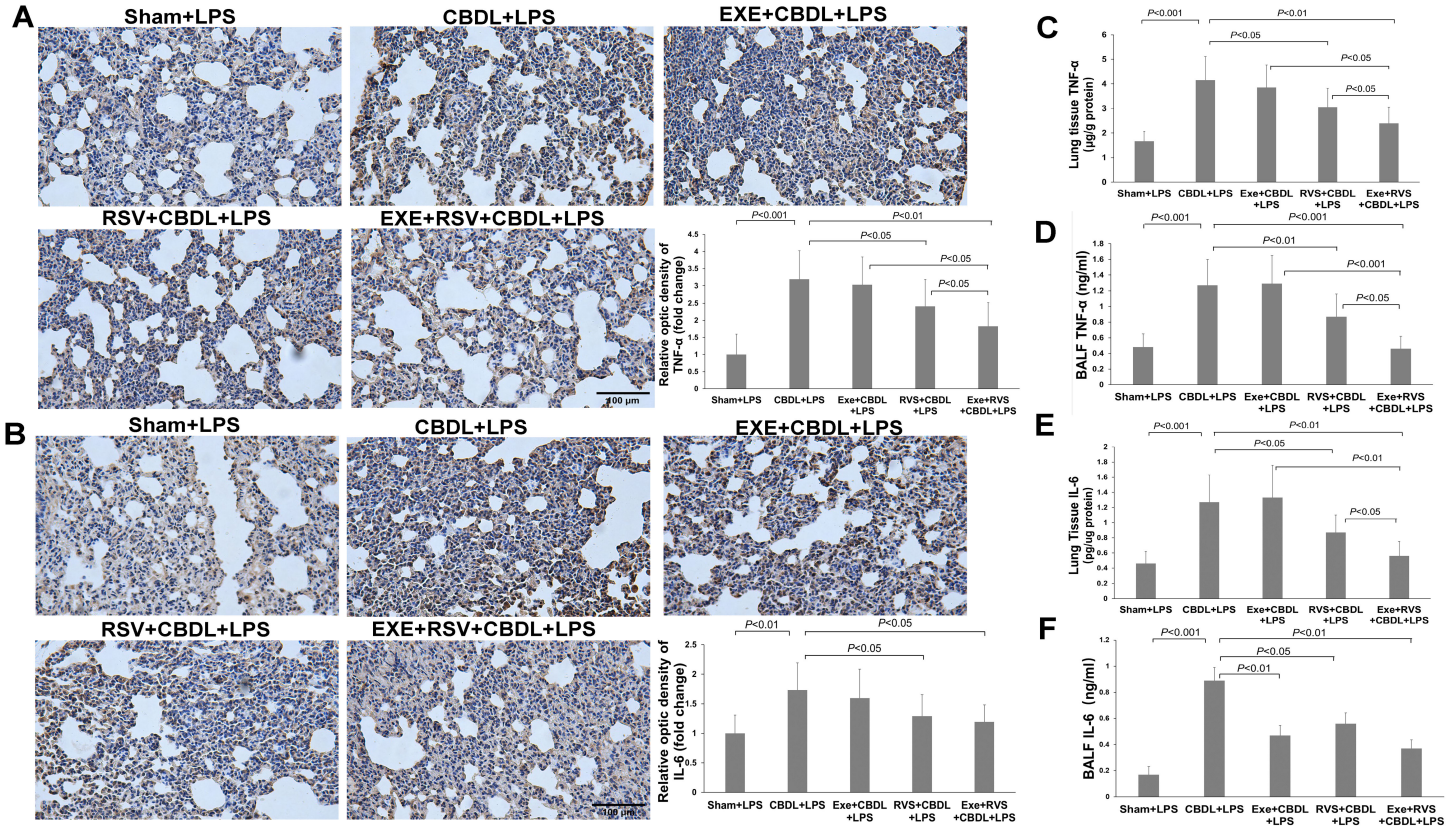
The effect of exercise and/or RSV on inflammatory cytokines (n=6). **(A)** Representative immunohistochemical image of TNF-α. **(B)** Representative immunohistochemical image of IL-6. **(C)** TNF-α levels in lung tissues. **(D)** TNF-α levels in BALF. **(E)** IL-6 levels in lung tissues. **(F)** IL-6 levels in BALF. Levels of TNF-α and IL-6 in **(C–F)** were measured by ELISA. Data analyzed by one-way ANOVA, LSD test for group comparisons. LPS, Lipopolysaccharide; CBDL, common bile duct ligation; Sham, Sham operation; RVS, resveratrol; EXE, exercise.

TNF-α and IL-6 are important inflammatory mediators in cholestatic lung injury. To further evaluate the excessive pulmonary inflammation induced by LPS in rats with obstructive jaundice, we observed changes in the expression of inflammatory cytokines TNF-α and IL-6 in lung tissue. Immunohistochemistry showed that compared to sham-LPS rats, the expression of TNF-α (**Figure 5A**) and IL-6 (**Figure 5B**) in the lung parenchyma of CBDL-LPS rats (especially in the pulmonary epithelial cells and their surrounding lung parenchyma) significantly increased, indicating a severe inflammatory response.

To further quantify these two cytokines, we performed ELISA on lung tissues and BALF. The results demonstrated a significant increase in TNF-α (**Figure 5C**) and IL-6 levels (**Figure 5E**) in the lung tissues of CBDL-LPS rats. Exercise training alone did not significantly reduce the levels of TNF-α and IL-6 compared to CBDL-LPS rats. However, the combination of exercise training and RSV significantly enhanced the reduction of RSV on TNF-α and IL-6, manifesting as a significant reduction in the levels of TNF-α and IL-6 with the combined intervention compared to animals receiving any single treatment (P<0.05).

Similarly, ELISA results in BALF revealed a significant increase in TNF-α (**Figure 5D**) and IL-6 (**Figure 5F**) levels in CBDL-LPS rats, which were significantly attenuated after appropriate intervention, especially the increase in IL-6. Moreover, RSV, exercise, and their combination also attenuated the increase in TNF-α. More importantly, compared to animals receiving any single treatment, combined intervention synergistically reduced the increase in TNF-α.

**Figure 6:**
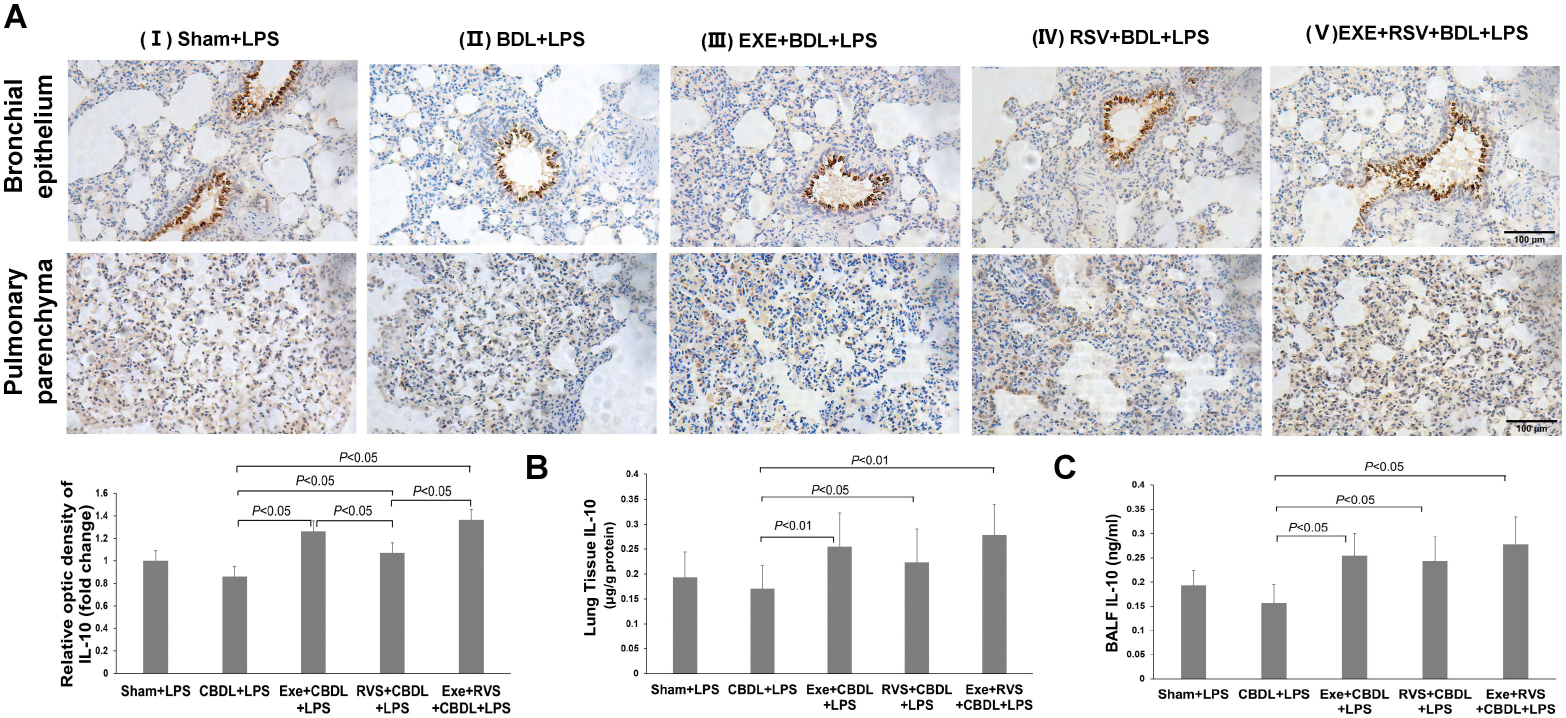
Effects of exercise and/or RSV on anti-inflammatory cytokines (n=6). **(A)** Representative immunohistochemical staining images of IL-10 in lung bronchial epithelium and parenchyma. **(B)** IL-10 content in lung tissue. **(C)** IL-10 content in BALF. Levels of IL-10 in **(B, C)** were measured by ELISA. Data analyzed by one-way ANOVA, LSD test for group comparisons. LPS, Lipopolysaccharide; CBDL, common bile duct ligation; Sham, Sham operation; RVS, resveratrol; EXE, exercise.

The anti-inflammatory effect of exercise can be partially achieved by increasing IL-10 expression. Therefore, we further evaluated the expression of IL-10 by using immunohistochemistry and ELISA. Immunohistochemistry analysis revealed that IL-10 was mainly distributed in the bronchial epithelium. Compared to sham-LPS rats, there was a decreasing trend in IL-10 expression in CBDL-LPS rats, but without significant difference. After the respective interventions, there was a significant increase in IL-10 expression, and both exercise and combined interventions demonstrated better effects compared to RSV intervention alone (**Figure 6A**). Furthermore, immunohistochemistry also showed that exercise significantly increased IL-10 expression in bronchial epithelium, while RSV appeared to induce a more notable increase in IL-10 within lung parenchyma (**Figure 6A**).

Further, ELISA results revealed a significant increase in IL-10 content in lung tissue (**Figure 6B**) and BALF (**Figure 6C**) after the respective interventions. Both exercise intervention alone and combined intervention showed equally good intervention effects in lung tissue.

### 3.6 The effect of rrIL-6 on lung injury in CBDL-LPS model rats

**Figure 7:**
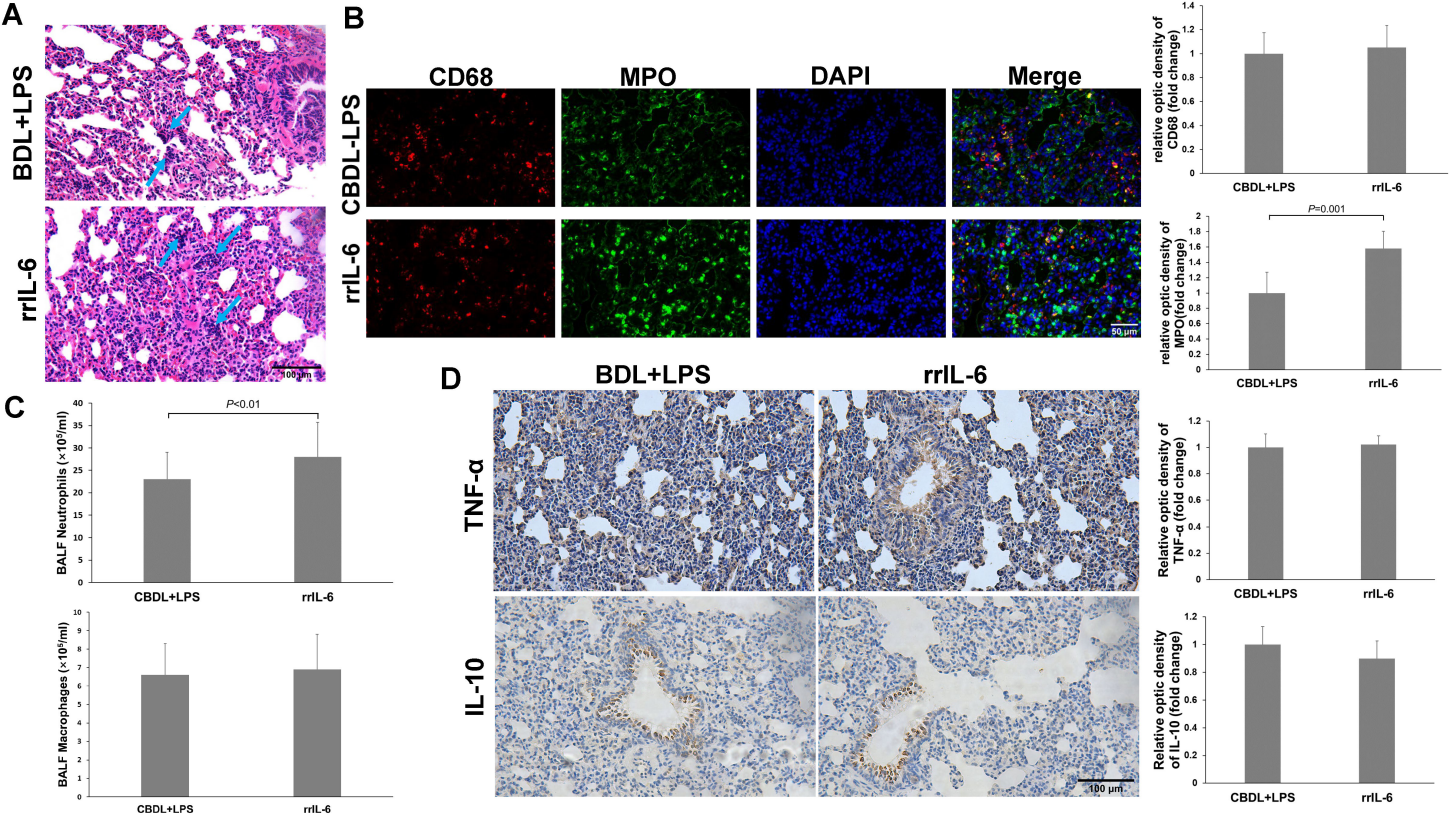
Effects of rrIL-6 on lung injury in CBDL-LPS rats (n=5). **(A)** Representative HE staining images showing the histopathological changes in lung tissues. **(B)**, Representative immunofluorescence images of MPO and CD68. **(C)** Neutrophil and Macrophage counts in BALF; The method is the same as above. **(D)** Representative immunohistochemical staining images showing the expression of TNF-α and IL-10 in lung tissue. Data were analyzed by t-test. MPO, Myeloperoxidase; LPS, Lipopolysaccharide; CBDL, common bile duct ligation; Sham, Sham operation; RVS, resveratrol; EXE, exercise.

IL-6 is a pleiotropic cytokine. To further confirm the role of IL-6 in CBDL-LPS-induced lung injury, we evaluated lung inflammation and injury in model rats after administration of rrIL-6. We observed that two out of seven model rats died within 4 h of rrIL-6 treatment (n=7). HE staining of lung tissues from the remaining rats indicated increased inflammatory response and lung injury (**Figure 7A**). Immunofluorescence staining for MPO and CD68 revealed a significant increase in MPO-positive staining (**Figure 7B**). Additionally, neutrophils in the BALF showed a marked increase (**Figure 7C**). Immunohistochemical analysis demonstrated that administration of rrIL-6 did not decrease TNF-α expression or increase IL-10 expression (**Figure 7D**). These results suggest that in the early stages of endotoxemia in biliary obstruction, IL-6 may promote inflammatory response and injury.

### 3.7 Systemic inflammation

**Figure 8:**
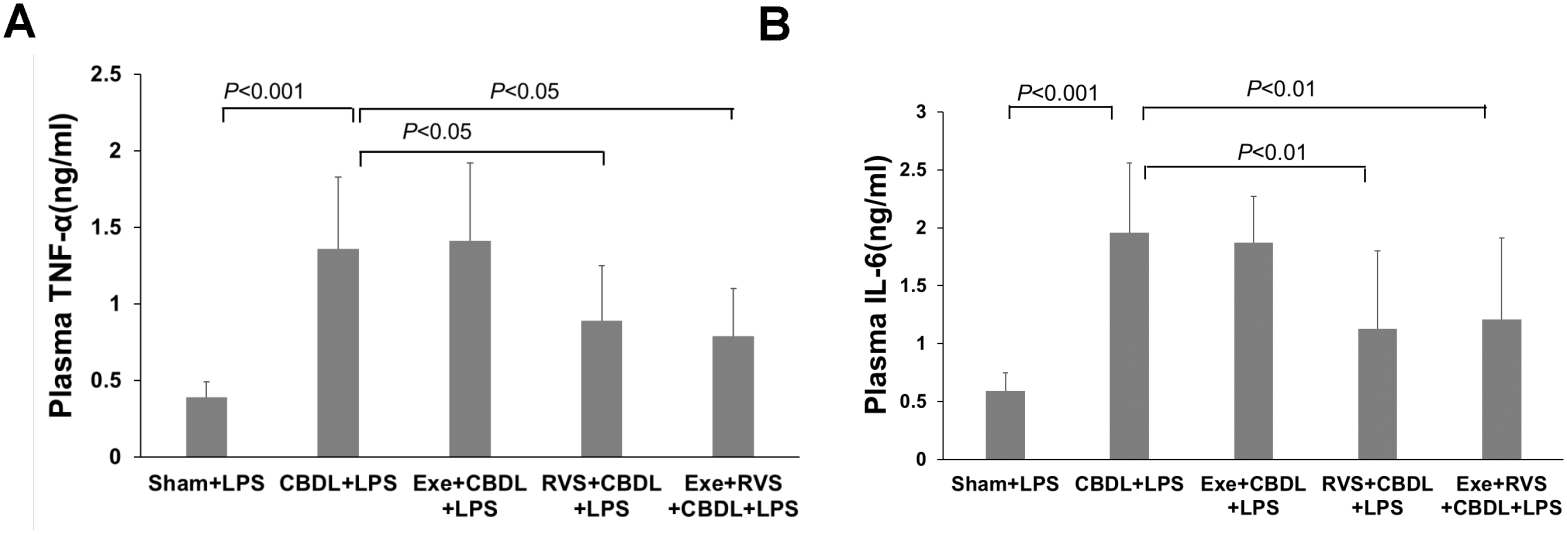
Effects of exercise and/or RSV on plasma TNF-α and IL-6 (n=6). **(A)** Plasma levels of TNF-α. **(B)** Plasma levels of IL-6. Levels of TNF-α and IL-6 in plasma were measured by ELISA. Data analyzed by one-way ANOVA, LSD test for group comparisons. LPS, Lipopolysaccharide; CBDL, common bile duct ligation; Sham, Sham operation; RVS, resveratrol; EXE, exercise.

Cholestatic lung injury is accompanied by the systemic inflammatory response, where the increased circulating TNF-α and IL-6 after biliary obstruction are important factors for excessive inflammatory reaction in the lungs. Therefore, we evaluated the plasma concentrations of TNF-α and IL-6. Compared to the sham-LPS group, administration of LPS significantly increased plasma TNF-α (**Figure 8A**) and IL-6 (**Figure 8B**) levels in CBDL-LPS rats, while RSV alone or in combination with exercise significantly alleviated this change.

## 4 Discussion

In this study, the CBDL-LPS rat model was considered successful based on the clinical manifestations of jaundice in rats, changes in ALT, AST, and blood bilirubin levels, as well as the high mortality rate and severe lung damage in CBDL rats after low-dose LPS administration. Administering LPS in the early stages of CBDL could more closely simulate the occurrence of endotoxemia after acute biliary obstruction. Similar to humans with obstructive jaundice, rats that develop jaundice after CBDL become highly sensitive to LPS and the resulting lung injury, increasing the lethal effects of LPS on the rats. At present, effective treatments for cholestasis-related lung injury are lacking in clinical practice. However, in this study, we further discovered that exercise preconditioning and/or RSV could significantly reduce the susceptibility of rats after CBDL to endotoxins through their anti-inflammatory effects, as evidenced by a reduction in excessive inflammatory responses, improvement in lung tissue pathological changes and edema, and extension of the survival time of the model animals. Additionally, the combined use of exercise and RSV demonstrated synergistic effects. These findings clearly provide valuable insights and novel strategies for the understanding and prevention of cholestasis-related lung injury in future clinical applications.

Excessive inflammatory response is the main pathological change in cholestatic animals after LPS attack, and it is also a major mechanism leading to lung injury during cholestasis (5, 6). The histological results in this study also indicate that after LPS administration, the lungs of CBDL rats showed obvious inflammatory cell infiltration and inflammatory reactions, accompanied by severe lung damage. Neutrophils and macrophages are the main immune cells in the inflammatory response in the lungs of CBDL rats, and their accumulation and activation in the lungs can produce a large number of inflammatory cytokines, reactive oxygen species, and proteases, causing serious damage to lung tissue (33). In this study, we found that even with a short duration of cholestasis in rats (4 days), a low dose of LPS induced the recruitment of a large number of macrophages and neutrophils in lung tissue, showing susceptibility to endotoxins. Exercise preconditioning combined with RSV intervention significantly reduced the infiltration of these inflammatory cells in the lungs and lung damage induced by LPS. Moreover, exercise training not only reduced the accumulation of neutrophils but also enhanced the inhibitory effect of RSV on the accumulation of macrophages in lung tissue, demonstrating a synergistic anti-inflammatory effect.

In addition to the accumulation of inflammatory cells, the inflammatory response also involves the secretion of many pro-inflammatory and anti-inflammatory factors. Among them, TNF-α, IL-6, and IL-10 play important roles in inflammatory responses of the lungs after cholestasis (5, 34, 35). TNF-α is a proximal inflammatory cytokine that initiates and maintains the inflammatory cascade. Besides directly damaging lung tissue, it can also aggravate the inflammatory cell accumulation and inflammatory responses by inducing the secretion of other inflammatory and chemotactic factors, ultimately leading to excessive inflammatory responses in the lungs and further tissue damage. IL-6 is a pleiotropic cytokine that acts downstream of TNF-α in the inflammatory cascade and can be induced by TNF-α. Like TNF-α, it can exacerbate the inflammatory cascade and the degree of tissue and organ damage, as well as increase mortality. In some cases, IL-6 is also considered to have anti-inflammatory effects (36), which are often closely related to stimulating the expression of IL-10 and inhibiting the production of TNF-α (37). IL-10 is an important anti-inflammatory cytokine in the inflammatory cascade and is also a crucial target for exercise to exert its anti-inflammatory effects (23).

In this study, the levels of TNF-α and IL-6 in the lung tissue of the model rats increased simultaneously without showing opposite changes. This was accompanied by severe lung tissue damage. Based on these results, we believe that TNF-α and IL-6 may exert promoting effects on inflammatory reactions and inflammatory damage in CBDL model rats. Notably, the lack of IL-6 enhanced the susceptibility of mice with obstructive jaundice to endotoxins and led to more extensive lung inflammation (38), which seems to suggest that IL-6 may have a protective effect. However, we found that after administering rrIL-6 to the model animals, lung tissue damage in rats significantly worsened and the mortality rate increased significantly. Interestingly, there was no significant decrease in TNF-α expression. Consistent with our findings, one previous study has also shown that after intraperitoneal administration of endotoxin, IL-6+/+ mice with cholestasis exhibited a significant inflammatory response, and although mice lacking IL-6 had relatively milder lung inflammation, they had similar degrees of lung tissue pathological damage (38). IL-6 is an important factor in causing similar injuries. Therefore, based on our research and previous reports, we speculate that an increase in IL-6, similar to IL-6 deficiency, increases the susceptibility of animals with obstructive jaundice to endotoxins and leads to excessive lung inflammation and damage. This also implies that at this stage, corresponding interventions are unlikely to exert anti-inflammatory effects by promoting IL-6 expression. Consistent with our speculation, our results found that exercise intervention did not increase the production of lung IL-6. On the contrary, it reduced the content of IL-6 in BALF and enhanced the effect of RSV on the reduction of TNF-α and IL-6 in lung tissue, and TNF-α expression in BALF. Compared with rats receiving any single treatment alone, combined intervention synergistically reduced the increase of TNF-α and IL-6, exerting an anti-inflammatory effect. Similarly, recent studies in sepsis models induced by intraperitoneal LPS injection and acute respiratory distress syndrome models have demonstrated that pre-exercise training can exert its protective effect by reducing the release of pro-inflammatory factors such as TNF-α and IL-6 (19, 23). However, a previous study on exercise in a lung injury model induced by intratracheal LPS instillation found that exercise could induce the expression of IL-6 in lung tissue and exert anti-inflammatory effects (39). Therefore, we believe that the protective effect of exercise through IL-6 is likely to depend on different disease backgrounds. Although there are differences in the understanding of IL-6 in different studies, both ours and previous studies consistently indicate that exercise can alleviate the inflammation and lung tissue damage induced by LPS.

In the inhibition of the inflammation cascade reaction in the lungs of CBDL-LPS model rats, another important finding of this study is that exercise and RSV also affected the expression of the anti-inflammatory cytokine IL-10. We found that IL-10 was mainly distributed in bronchial epithelial cells. After intraperitoneal injection of the same dose of LPS, cholestatic rats showed a decreasing trend in IL-10 expression compared to sham rats, although this trend did not have statistical significance. Previous studies have shown that in the early stages of the inflammatory cascade reaction, there is usually a competitive expression of IL-10 and pro-inflammatory cytokines, which determines the severity of inflammation, tissue damage, and mortality (40, 41). Therefore, we believe that the results of IL-10 in this study can still suggest that LPS and biliary obstruction may have a cumulative effect on the anti-inflammatory capacity of rat lungs. After the corresponding intervention, both exercise alone and in combination with RSV significantly increased the expression of IL-10, especially after exercise intervention, which better increased the expression of IL-10 in bronchial epithelial cells compared to RSV. IL-10 can reduce inflammatory reactions by inhibiting the release of inflammatory cytokines TNF-α and IL-6 (23). Based on these findings and the contrasting alterations observed in TNF-α, IL-6, and IL-10 in our study, it is plausible to suggest that the anti-inflammatory effects of both exercise and RSV interventions may be partly attributed to the upregulation of IL-10 expression.

The mechanism of inflammatory cell accumulation and activation in the lungs after cholestasis has not been fully elucidated at present. However, studies have shown that strong and persistent systemic overproduction of TNF-α and IL-6 is an important factor (13, 42). In rodent studies, excessive production of circulating TNF-α can induce the accumulation of macrophages and neutrophil infiltration in the pulmonary vasculature after CBDL (42, 43). Particularly, after endotoxemia, animals with obstructive jaundice often exhibit more intense systemic and pulmonary production of TNF-α and IL-6, as well as increased accumulation and activation of inflammatory cells (13, 43). Moreover, as their plasma concentrations increase, the degree of inflammatory cell accumulation and activation in lung tissue also increases (13, 14, 42). The drastic increase in circulating levels of TNF-α and IL-6 following LPS administration may partially explain why jaundiced animal lungs are more susceptible to LPS attack while corresponding antagonism can inhibit the accumulation of inflammatory cells in the lungs, alleviate excessive inflammatory reactions and lung damage, indicating an improvement in susceptibility. In this study, we found that RSV and its combination with exercise significantly reduced the overproduction of TNF-α and IL-6 in the rat plasma. This can partly explain the inhibitory effect of the corresponding intervention on the accumulation of pulmonary macrophages and massive infiltration of neutrophils in the model animal. This also implies that the corresponding intervention reduced the susceptibility of jaundiced animals to endotoxins. In addition, exercise training alone reduced the accumulation of neutrophils in lung tissue and exerted a synergistic anti-inflammatory effect with RSV in lung tissue without reducing the levels of circulating TNF-α and IL-6. On one hand, this might be related to their direct effects on the lungs. On the other hand, the infiltration of inflammatory cells involves many steps and influencing factors, and the effects of exercise on these need further analysis in the future. This is also a potential limitation of this study. Furthermore, exercise training failed to act synergistically with RSV to reduce the levels of circulating TNF-α and IL-6, suggesting that RSV intervention may independently reduce these inflammatory cytokines in circulation. Additionally, their combination cannot alleviate biliary obstruction, which may be another reason why they do not further reduce circulating TNF-α and IL-6 when used in combination. Given these intriguing results, we believe that further research is needed to clarify the beneficial roles of exercise and RSV in the specific clinical context of this disease, which will promote their future clinical applications.

## 5 Conclusion

In summary, we demonstrated that even in the early stage, obstructive jaundice increased the susceptibility of rats to LPS, making lung inflammation and injury more prominent in CBDL rats. Exercise and RSV could alleviate lung injury during endotoxemia in rats with obstructive jaundice and reduce the risk of mortality.

## Data Availability

The original contributions presented in the study are included in the article/supplementary material. Further inquiries can be directed to the corresponding author.
